# Studying Proton Mobility in Zeolites by Varying Temperature Infrared Spectroscopy

**DOI:** 10.3390/molecules24173199

**Published:** 2019-09-03

**Authors:** Pit Losch, Hrishikesh Joshi, Niklas Stegmann, Olena Vozniuk, Wolfgang Schmidt

**Affiliations:** Max-Planck-Institut für Kohlenforschung, Department of Heterogeneous Catalysis, 45470 Mülheim an der Ruhr, Germany (H.J.) (N.S.) (O.V.)

**Keywords:** pProton-conducting materials, VTIR, acidic materials, zeolites

## Abstract

We report a varying temperature infrared spectroscopic (VTIR) study with partial deuterium isotopic exchange as a method for characterizing proton mobility in acidic materials. This VTIR technique permits the estimation of activation energies for proton diffusion. Different acidic materials comprising classical proton-conducting materials, such as transition metal phosphates and sulfonated solids, as well as different zeolites, are tested with this new method. The applicability of the method is thus extended to a vast library of materials. Its underlying principles and assumptions are clearly presented herein. Depending on the temperature ranges, different activation energies for proton transfer are observed irrespective of the different materials. In addition to the well-studied transition metal phosphates, Si-rich zeolites appear to be promising proton-transfer materials (with E_act_ < 40 kJ mol^−1^) for application in high-temperature (>150 °C) PEM fuel cells. They significantly outperform Nafion and sulfonated silica, which exhibit higher activation energies with E_act_ ~ 50 and 120 kJ mol^−1^, respectively.

## 1. Introduction

Initially, we were motivated to characterize the acidity of zeolites. Achieving an reliable experimental and theoretical characterization of zeolites intrinsic acid strength as an isolated parameter remains challenging. Acid strength characterization often relies on weakly basic probe molecules [1]. However, the unbiased characterization of zeolite acid strength, independent of overlapping potentials caused by similar pore sizes and molecular diameters, also known as surface curvature, remains unachieved. 

Unlike transition metal phosphates (TMPs) or sulfonated solids, which both have direct acidic groups [2,3], the Brønsted acidic character of zeolites results from trivalent atoms (mostly Al but also B, Ga, or Fe) substituting silicon atoms in tetrahedral positions in a crystalline silicate framework. This substitution leads to local negative charges compensated by metal cations or protons. It is still debated whether or not these Brønsted bridging (Si-(OH)-Al) acid sites (BAS) might exhibit different intrinsic acid strengths depending on their concentration and the local chemical environment (i.e., spatial separation to the nearest Al site); zeolite topology-related parameters, such as Si-(OH)-X bond lengths and angles; and, finally, the nature of X itself (with Si-(OH)-X, where X = B, Al, Ga, Fe etc.) [4,5]. These hypotheses have been extensively studied with the advent of increasingly sophisticated spectroscopic and theoretical techniques, yet it is still difficult to gather independent parameters describing zeolites acidity [6,7,8].

Fundamental insights in zeolites acidity were obtained by ^1^H MAS NMR [9,10], infrared spectroscopy with or without probe molecules, and thermally programmed desorption of weakly basic probe molecules (B-TPD) [11,12,13,14,15,16,17]. However, techniques relying on probe molecules typically reflect dispersion and polar interactions due to their surface curvature and the consequent confinement effect between adsorbed molecules and pores [18]. In contrast, typical proton-conducting materials, such as TMPs and sulfonated solids, are not microporous and normally do not exhibit such confinement effects [19,20,21].

Information on the intrinsic acid strength or the O-H bond weakness of zeolitic BAS is thus useful for the description of the zeolite acid character independent of their pore topology. We are convinced that such independent characterization may enable multi-parametric analysis and the optimization of zeolite materials used in, for example, catalysis.

Interestingly, impedance spectroscopy at different temperatures with varying relative humidity can be directly used to determine proton conductivity, which can be characterized by activation energy for a translational proton transfer between two BAS-sites within zeolites. At very low relative humidity, this proton transfer has to occur as single proton hopping, where the proton can interact during the hopping with the potential of the pore walls. In our opinion, proton conductivities have to relate to the intrinsic acid strength in zeolites [22,23].

Here, we present in detail our recently reported model relying on an isotopic H/D exchange infrared spectroscopic study with temperature variation (VTIR) [24]. The partial H/D exchange allowed for the use of an adapted VTIR model to estimate proton transfer activation energies in different temperature ranges (ambient −150 °C and 150–250 °C). It is shown that Si-rich zeolites may be promising proton transfer materials for high-temperature (>150 °C) fuel cell applications, while we also applied this method to classical proton-conducting materials like TMPs and sulfonated solids.

## 2. Results

### 2.1. Setup

A set of standard materials for proton conduction membranes, such as sulfonated solids (Nafion and SBA15-SO_3_H) and TMPs (ZrP_2_O_7_ and TiPO_4_), have been studied using the newly developed method relying on VTIR. The extracted activation energies for proton transfer are compared to various commercial acidic zeolites, including ZSM-5 (Si/Al = 13.5; 45 and Si/Fe = 45), Beta (Si/Al = 12.5) and USY (Si/Al = 15, meso-15).

In Table 1, textural properties such as apparent BET surface areas (S_BET_), micropore volumes (V_µ_) (if applicable) and acid site densities (n(H^+^)) of the tested materials are reported.

Our experimental setup, as explained in further detail in the experimental part, is based on a DRIFT cell. Experimental details concerning the used setup have also been reported in our previous work [24]. In short, all samples were activated in the cell under dry N_2_ flow at 250 °C before the H_2_O:D_2_O (1:1) mixture (formal HDO) with a partial pressure of 3.16 kPa, namely, H_2_O vapor pressure at 25 °C in N_2_ flow (~10 mL min^−1^) was fed into the cell. It is important to note that in this work the activation energies for proton transfers were studied at relatively low relative humidity. The partial pressure of partially deuterated water remained constant at p°(HDO) = 3.16 kPa throughout the whole temperature dependent measurements. Equilibrium of ongoing isotopic exchange was reached in all cases after 60 min. The cell was then closed and spectra were acquired at different temperatures by gradually lowering the temperature, usually in steps of 10 °C, while keeping the atmosphere unchanged.

### 2.2. IR Spectra at Varying Temperatures

**Figure 1:**
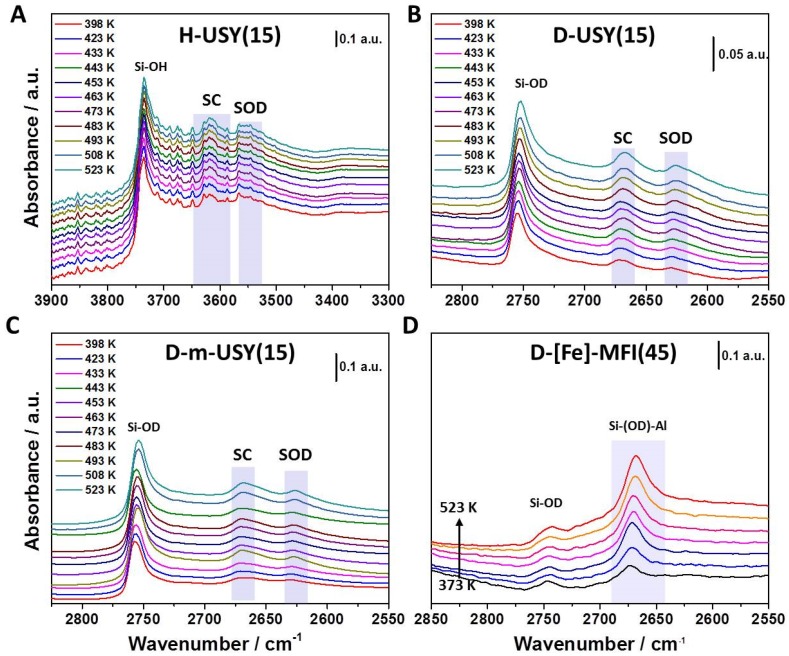
Varying temperature infrared spectroscopic (VTIR) spectra for H/D exchange for (**A**) O-H stretching region of H-USY(15), (**B**) O-D stretching region for D-USY(15), (**C**) D-m-USY(15), and (**D**) D-[Fe]-ZSM-5(45). To determine peak positions and to integrate relative signal intensities, a second derivative baseline was used. Absolute integrated areas were used in the following data treatment.

Figure 1 is presenting typical series of temperature dependent IR spectra in the OH (Figure 1A) and OD (Figure 1B–D) stretching region (2400–2900 cm^−1^). For all studied samples, the signal intensity for the stretching band of deuterated Brønsted acid sites increased in line with increasing temperatures, whereas the OH stretching signal increased for decreasing temperatures. We showed in our previous study that this apparent population change cannot be linked to altered populations of rovibrational states [28]. We observed that the first overtone signal for BAS(OD) showed a decrease in signal intensity from 75–150 °C, but the difference in intensity was significantly lower than the *Δ*A_OD_(T) observed at 2400–2900 cm^−1^. The observed intensity loss of BAS(OD) bands with lower temperature is general for the whole series of analyzed samples.

### 2.3. Adapted VTIR Method for the Determination of the Activation Energy for Proton Transfer

**Figure 2:**
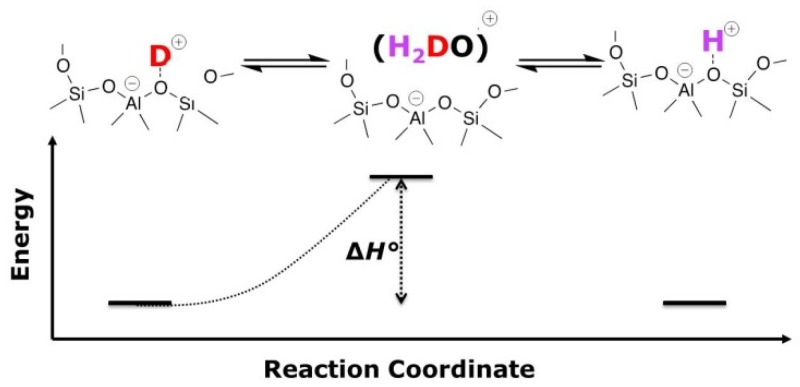
Illustration of a sequence of equilibria, of which the first half reaction is considered.

The derivation of the VTIR equation used in this study is inspired by E. Garrone and C.O. Arean [14,29], but does not use the van’t Hoff equation because the aim is not the characterization of adsorption processes. Instead, as it is represented in Figure 2, we consider the elementary steps in our system. The process of an H/D exchange is considered as a sequence of two reactions. With this assumption, and also based on the fact that at sufficiently high temperatures, i.e., the herein studied temperature ranges, protons of all acidic OH groups must be in rapid exchange, we now only consider the first half reaction herein. In short, the disappearing signal intensity of the O-D stretching band is monitored.

Thus, we have to assume that the O-D bond breaking event is a temperature-dependent equilibrium.

(1) ΔG°=ΔH°−TΔdS°

(2) $$\Delta G{}^{\circ}=-R\;T\;ln\left(K_{eq.}\right)$$

(3) $$ln\left(K_{eq.}\right)=\frac{\Delta S{}^{\circ}}{R}-\frac{\Delta H{}^{\circ}}{R\;T}$$

Equation (3) can be written in terms of change in absorbance. Since we focus on changes in one single band, typically applied data correction methods, such as *Kubelka Munk (KM)*, are not necessary. Nevertheless, it has been confirmed that KM corrected data also led to the same apparent activation enthalpy values.

(4) $$\ln\left(\frac{A}{A_{max@250\;{}^{\circ}C}-A}\right)=\frac{\Delta S{}^{\circ}}{R}-\frac{\Delta H{}^{\circ}}{R\;T}$$

As outlined above, a bond breaking and forming (H/D-exchange) event is observed in this study; therefore, the VTIR model is adapted permitting the evaluation of the energy required for (H/D)^+^-diffusion.

The adapted VTIR-equation (4) plots the natural logarithm of the relative population of D-BAS sites at a certain temperature, given as the ratio between absorbance (A) on one side and the difference between the maximum population (*A_max_@523 K*) at 523 K and the considered absorbance (A), as a function of temperature (*T*^−1^), on the other. Data were extracted by integrating the v_OD_ stretching bands of Brønsted acid sites. The underlying chemical process of the considered phenomenon is the D-BAS bond breaking. The apparent activation energy of the proton transfer E_act_ can then be approximated to *ΔH*°, which can be extracted by linear regression of the graphical results. Though the slopes (*ΔH*° *R*^−1^) of these lines correlate with E_act,_ it can be noted that the intercept (*ΔS*° *R*^−1^) relates to the apparent activation entropy (*ΔS*° ≈ *ΔS*_act_) of this exchange. Apparent activation entropy values at temperatures 423–523 K exceed 100 J (mol K)^−1^, while at 348–423 K *ΔS*_act_ they remain below 50 J (mol K)^−1^. Therefore, the observed transformation is likely to happen in a gaseous phase (high *ΔS*_act_) at high temperatures, whereas it seems to occur in a more condensed state (low *ΔS*_act_) at lower temperatures.

**Figure 3:**
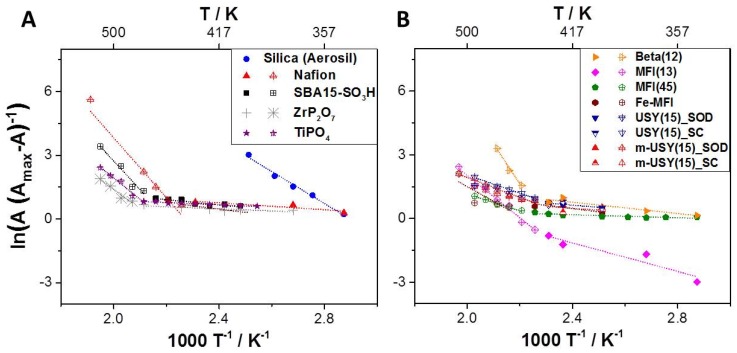
(**A**) VTIR plots for H/D-exchange on different typical proton-conducting materials and (**B**) for different zeolites with different topologies, Si/Al ratios, and micro–mesoporosity.

Considering the plotted data in Figure 3, it appears that different apparent activation energies are obtained in different temperature ranges. These ranges are defined as follows: low temperature (LT): 348–423 K and high temperature (HT): 423–523 K. This observation is indicative for two distinct proton transfer mechanisms.

Apparent activation energies for H/D exchange that were determined from the plots (Figure 3) suggest two different H/D exchange mechanisms depending on the temperature ranges. In agreement with literature, at high temperatures higher apparent activation energies are needed for proton diffusion due to low levels of hydration, even at high relative humidity. Low activation energies are observed at low temperatures with higher relative humidity. At lower temperatures, the partial pressure of water p°(HDO) = 3.16 kPa becomes a significant parameter. In the well-studied case of Nafion, proton transfer activation energies have been linked to different mechanisms. E_act._ between 10 and 20 kJ mol^−1^ are reported for high hydration levels and up to 30–40 kJ mol^−1^ for lower hydration levels [2].

**Figure 4:**
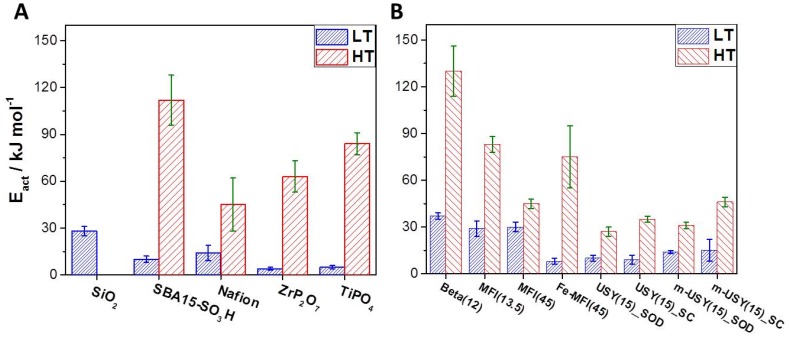
Apparent activation energies for proton transfer with experimental errors plotted for different materials. Values for low-temperature ranges are in blue (348–423 K), and values for elevated temperatures (423–523 K) are in red. (**A**) Data for classical proton-conducting materials and (**B**) data for different zeolites. Several silicon rich zeolites exhibit similar or even lower activation energies for proton transfer as TMPs which are commonly used for high-temperature PEM fuel cells.

Our method was first applied to Nafion, SBA15-SO_3_H, and TMPs, which led to values agreeing with data from literature. These results are plotted in Figure 4A, while data for different zeolites are shown in Figure 4B. It can be seen that silicon-rich samples exhibit proton mobility close to that of Nafion, which is a standard material for proton exchange membranes for low-temperature fuel cells. High-temperature PEM fuel cells often rely on ZrP_2_O_7_ and TiPO_4_ as material for proton conduction. These have also been studied with this novel method. The obtained activation energies are again in good accord with reported data [19]. These TMPs are used at temperatures of >150 °C because Nafion fails as a proton conductor under these conditions. Interestingly, while non-acidic amorphous silica is not a good proton conductor at elevated temperatures, it appears that sulfonic acid groups grafted into mesoporous silica SBA15 significantly improve proton conduction at high temperatures. It has been reported that the studied SBA15-SO_3_H material is synthesized in several steps in a controlled way and that it can withstand temperatures of up to 300 °C [25].

Astonishingly some zeolites, especially silicon-rich samples like ZSM-5(45) and m-USY(15), showed activation energies for proton conduction < 45 kJ mol^−1^. It is interesting to see that for [Fe]-ZSM-5 samples instead of [Al]-ZSM-5, the activation energy was much higher. Thus, as discussed previously, the nature of the trivalent heteroatom seems to have a significant influence on the proton conduction. On the other hand, the mesostructured USY sample with hierarchical porosity does not exhibit any enhanced proton conductivity, i.e., lower activation energy. Concerning USY samples, it was possible to evaluate discrete activation energies for proton transfer occurring in the SOD cages and in the supercages (SPC) from the discrete signals at 2620 and 2670 cm^−1^ respectively.

## 3. Discussion

In this work, two mechanisms of proton transfer were observed independent of the type of material. Considering existing reports on well-studied materials, it appears reasonable, also for zeolites, to attribute the low-temperature mechanism to a water-chain mediated Grotthus-like (quasi-)transport, while at high temperatures a vehicular transport mechanism with the presence of diffusing hydronium ions or clusters (H_2n+1_O_n_^+^) is in effect. In absolutely dry conditions, direct intra-crystalline proton hopping along micropores can be imagined, though under the studied conditions verification of this process is experimentally impossible.

The above mechanistic hypotheses are supported by numerous reports in the literature suggesting that zeolites at 348–423 K retain one to three water molecules at their acid site, and thus may still contain H_7_O_3_^+^ trimers, H_5_O_2_^+^ dimers, and H_3_O^+^ cations up to 423 K [30,31]. Alberti and colleagues reported a neutron diffraction study indicating that residual H_2_O participates in the H/D-exchange at the level of the acid sites [32]. Kreuer et al. conducted a theoretical study on H and D transfer, and they concluded that ideal proton conductors are characterized by ‘soft’ oxygen species or weak O-H bonds [33]. It is therefore reasonable to assume that proton conductivity for solid acid materials can be improved by increasing intrinsic acid strength.

Materials studied as potential proton conductors for construction of high-temperature PEM fuel cells are often very expensive. The tested materials include perovskites [34], layered oxides [35], mesoporous silica with grafted sulfonic acid groups [36], graphene oxides [37], and metal organic frameworks (MOFs) [38]. Growing interest in these materials has led to the development of different standard procedures for measurement of proton diffusion. Impedance spectroscopy and ^1^H MAS NMR at different temperatures are commonly used methods. Proton conductivity on zeolites investigated by means of complex impedance spectroscopy support our findings. Franke and colleagues determined activation barriers for proton transport in zeolites between 39–49 kJ mol^−1^ in the temperature range 373–473 K and 74–77 kJ mol^−1^ for the temperature range of 573–773 K [22,23]. Ryder et al. calculated activation energies for proton hopping in ZSM-5 zeolites in the temperature range 200–1000 K [39]. Considering the assistance of water in the proton exchange mechanism, they report activation energies as low as 8–30 kJ mol^−1^ varying with the chosen model. They also analyzed completely dry proton hopping on a theoretical basis, for which 90–150 kJ mol^−1^ activation energy was established.

In agreement with the preceding work cited above, our study suggests that zeolites are a promising alternative to TMPs for the construction of high-temperature PEM fuel cells.

In fact, zeolites have been used as membrane material for methanol fuel cells [40]. Given the interest in designing high-temperature proton-conducting membranes, this work describes a cheap method to characterize proton conductivity [41,42]. Furthermore, we show that Si-rich zeolites should also be considered for high-temperature proton membrane applications.

Lastly, one could argue that even though zeolites may conduct protons, their porosity makes them prone to crossover of gases. In this regard, we indicate that various techniques are available to fill the micropores of zeolites to make them impermeable to gases. Usually used for hard templating [43], a graphite@zeolite hybrid may actually offer interesting proton and electron conduction properties.

## 4. Materials and Methods

### 4.1. Materials

Commercially available zeolite samples were investigated: ZSM-5(Si/Al = 13 and 45), beta (Si/Al = 13 and 75), and USY (Si/Al = 15) were obtained in their ammonium form from Süd Chemie (now Clariant, Munich, Germany), Degussa (now Condea, Midrand, South Africa) and Alfa Aesar (Haverhill, MA, USA). These materials were calcined at 823 K for 8 h (5 K min^−1^ ramp) and then used in the DRIFT setup. One [Fe]-ZSM-5 with a nominal Si/Fe ratio of 45, has been synthesized by replacing the Al-source in a reported fluoride mediated zeolite synthesis [17] by Fe(NO_3_)_3_ x 9 H_2_O. The mesostructuring treatment of USY(15) has been performed as previously reported [27].

In addition, terminal silanol-rich amorphous silica (AEROSIL 200), strongly acidic polymer Nafion (Aldrich, Darmstadt, Germany), a sample with sulfonic acid groups grafted on SBA15-SO_3_H, synthesized following a reported procedure [25], and two TMPs, namely reported crystalline phases of ZrP_2_O_7_ and TiPO_4_ have also been analyzed herein.

### 4.2. Methods

Infrared spectra were collected in DRIFTS mode using 2–5 mg sample and a modified Harrick DRIFTS cell with KBr windows. Spectra were measured in the 4000–1000 cm^−1^ range with a 4 cm^−1^ resolution and averaging 250 scans using a Nicolet Magna-IR 560 spectrometer equipped with a MCT detector (Thermo Nicolet, Waltham, MA, USA). Samples were dried at 523 K under flowing dry nitrogen (10 mL min^−1^) for 30 min. After this in situ activation, the collection of spectra was started. All spectra were normalized by the intensity of the Si–O–Si overtones (2100–1750 cm^−1^).

Isotope exchange experiments were performed on activated samples. The vapor pressure (3.16 kPa) at 298 K of a 1:1 mixture of H_2_O:D_2_O was used as a feed for the experiments. After 60 min of exchange, the cell was closed and temperature-dependent series of spectra were acquired starting from 523 down to 348 K.

Complementarily to the novel acidity evaluation in this study, the samples were evaluated with ammonia temperature programmed desorption (NH_3_-TPD). NH_3_-TPD was performed on a Micromeritics Autochem II 2920 device. 100 mg of catalyst was activated at 773 K for 1 h (heating ramp of 5 K min^−1^) and then cooled to 323 K. The sample was exposed to a flow of 10% NH_3_/He for 30 min and subsequently purged in helium for 2 h. The desorption profile was collected in the range of 373 to 1073 K with a heating rate of 10 K min^−1^. The corresponding acid site densities are listed in Table 1.

Micropore volumes and apparent specific surface areas were measured by means of N_2_ physisorption. These measurements were carried out on a Micromeritics 3 Flex instrument. First, the samples were degassed for 8 h at 513 K under vacuum using a Micromeritics Smart VacPrep unit. Sorption isotherms were then collected at 77 K using a static volumetric method. External surface area, mesopore, and micropore volumes of the materials were determined via t-plot analysis performed using the 3 Flex software package.

## 5. Conclusions

The development of a varying temperature infrared spectroscopic (VTIR) method with partial isotopic deuterium exchange for the determination of proton transfer activation energies has been described. Different zeolites were tested, and this VTIR technique showed low activation energies for proton diffusion for silicon-rich zeolites. Si-rich zeolites compete with classical proton-conducting materials such as transition metal phosphates (TMPs) and sulfonated solids. Depending on the respective temperature ranges (<423 vs >423 K), different activation energies were observed for all different materials suggesting two different proton conduction mechanisms. Even at temperatures above 423 K (150 °C), Si-rich zeolites exhibited E_act_ < 40 kJ mol^−1^, making them attractive for the application in high-temperature PEM fuel cells. In these high-temperature conditions, they significantly outperform Nafion, transition metal phosphates, and sulfonated silica, with E_act_ ranging from 40, 60 to 120 kJ mol^−1^, respectively.

## Figures and Tables

**Table 1:** Characteristics of tested materials.

Entry	Samples	S_BET_/m^2^ g^−1^	V_µ_/cm^3^ g^−1^	n(H)/µmol g^−1 c^
1	Nafion	<30	0	n.d.
2	Silica	187	0.01	0
3	SBA15-SO_3_H ^a^	431	0.82	900 ^d^
4	ZrP_2_O_7_	10	0	29
5	TiPO_4_	16	0	119
6	Beta(12.5)	555	0.18	502
7	ZSM-5(13.5)	439	0.17	1021
8	ZSM-5(45)	415	0.16	257
9	[Fe]-ZSM-5(45)	298	0.13	~300
10	USY(15)	778	0.25	900 ^e^
11	m-USY(15) ^b^	874	0.16	887 ^e^

^a^ Synthesized as reported previously in [25]; ^b^ synthesized as reported elsewhere [26,27]; ^c^ acid site densities were determined with NH_3_-TPD unless stated otherwise; ^d^ acid site density determined by titration with 0.1 M NaOH after equilibrating the sample with 2 M NaCl; and ^e^ H_2_O-TPD data from our previous study were used [27].
